# The Effect of Feeding Strategies Based on Flaxseed and Locally Available Plant-Derived Additives on the Growth Performance, Biochemical and Immune Blood Indices, and Antioxidant Capacity of Pigs in Sustainable Production

**DOI:** 10.3390/ani16142236

**Published:** 2026-07-19

**Authors:** Małgorzata Świątkiewicz, Magdalena Szyndler-Nędza, Anna Czech, Kinga Szczepanik, Mirosław Tyra, Marek Babicz

**Affiliations:** 1 Department of Animal Nutrition and Feed Science, National Research Institute of Animal Production, ul. Krakowska 1, 32-083 Balice, Poland; kinga.szczepanik@iz.edu.pl; 2 Department of Pig Breeding, National Research Institute of Animal Production, ul. Krakowska 1, 32-083 Balice, Poland; magdalena.szyndler@iz.edu.pl (M.S.-N.); miroslaw.tyra@iz.edu.pl (M.T.); 3 Department of Biochemistry and Toxicology, University of Life Sciences in Lublin, ul. Akademicka 13, 20-950 Lublin, Poland; anna.czech@up.edu.pl; 4 Department of Animal Breeding and Agricultural Consulting, University of Life Sciences in Lublin, ul. Akademicka 13, 20-950 Lublin, Poland; marek.babicz@up.edu.pl

**Keywords:** pig feeding, flaxseed, dried herbs, dried fruits, health status, oxidative stability, blood indices, sustainable pig production, native pigs

## Abstract

The study evaluated the effects of feeding strategies on growth performance, health laboratory biomarkers, and antioxidant protection of slow-growing pigs, fed finisher diets containing alternative protein sources (local and by-product), as an element of sustainable pig production. The study involved 40 fattening pigs (Pulawska native breed), fed the same grower mixture, while dietary treatment was applied during the finisher phase (90–135 kg BW). The diet in all groups was isoenergetic and isoprotein, but differing in additives. Control pigs—mixture without additives; experimental groups—feed supplemented with 3% flaxseed together with 2% dried herbs (H), or 2% dried fruit (F), or 2% herbs with dried beetroot (HB). The experimental finisher diets did not change the fattening results, carcass, and physico-chemical traits of meat quality. Summarized, the tested feeding strategies can be proposed for the production of native (local) pork with higher PUFA n-3 and dietetic added value combined with the oxidative stability of the meat, with the diet containing flaxseed together with dried herbs proving particularly effective for inhibiting the negative effects of oxidative stress in pigs (higher CAT, SOD, GSH and lower MDA, LOOH observed in blood and meat, *p* ≤ 0.05), and improving their immune blood indices (higher IgA, IgG, IgM, lysozyme, lower Il6 and Il8, *p* ≤ 0.05).

## Simple Summary

Modern pig production should be characterized by sustainability, which is a complex concept. We focused on broadly defined local aspects, reinforcing the link between sustainability and territory exploration, while encompassing the native pig breed and novel feed sources (including by-products), which demonstrates care for pigs’ growth efficiency and health, and the high quality (dietetic) of animal products. The novelty of our research is the use of a composition of local natural sources of n-3 PUFAs together with local natural antioxidants, introduced to feed containing alternative protein sources intended for native pig breeds, in order to protect the health status of pigs used for producing high-PUFA n-3 dietary meat. The results obtained showed that the tested feeding strategies can be proposed for the sustainable production of native (local) pork with dietetic added value combined with the oxidative stability of meat, with the diet containing flaxseed together with dried herbs proving particularly effective.

## 1. Introduction

Modern pig production should be characterized by sustainability, which is a complex, multicomponent, interactive process, with a broad concept encompassing many areas of action [1]. Among other goals, maximizing growth efficiency should be combined with reducing negative environmental impact, as well as the animals’ welfare, avoiding antibiotic treatment, and biosecurity, which should be taken into account. It is also desirable to consider broadly defined local aspects, which help reduce the carbon footprint of transport origins. One of the directions of sustainable pig production is achieving higher quality and value (nutritional, dietetic) of animal products. Sustainable feeding strategies include optimizing feed formulations in different breeds and management conditions, improving feed efficiency, exploring novel feed sources (including by-products), improving the quality of animal-derived products, and reinforcing the link with the territory [2].

Our tactic proposed in the presented manuscript is a combination of nutritional factors with the trend of supporting locality and by-product inclusion, which may be an option for sustainable pig production. The choice of a native breed of slow-growing pigs is dictated by the current trend of consumers demanding meat from pigs produced under more extensive conditions, providing animals with more space, environmental enrichment, more aspects of animal welfare, and a varied diet with foraging opportunities [3,4,5]. Slow-growing pigs have a lower nutrient requirement, so feed concentration can be reduced, which is important because feed constitutes the highest cost in pig farming and remains a critical factor influencing its profitability. It is easier to introduce local protein sources, such as legume seeds or by-products such as press cakes, into the diet of pigs with lower nutrient requirements, because these materials contain high fiber content, which is not desirable in the diet of high-meat pigs of fast-growing breeds. Another important element of sustainable animal production is the feeding strategy, which should improve both animal health prevention through feed additives, as it contributes to reducing the use of therapeutic antimicrobials [6], and the quality of animal products. Our feeding concept is not only based on the use of local sources of protein and energy (including natural sources of n-3 PUFAs) to reduce reliance on conventional raw materials and increase crop biodiversity, but also assumes the incorporation of functional ingredients and bioactive substances that can support feed utilization, enhance gut health, or improve meat quality.

Nowadays, consumers’ growing interest is directed towards information about production methods to make informed purchase choices, and consumers rely more on extrinsic quality attributes in their decision process [7]. Extrinsic cues are mainly related to production, processing, and marketing, and include animal breed, feeding, resources, brand, price, packaging, certificates, manufacturer, and labeling—welfare, environmental, sustainability, organic, etc. [8,9]. Such attributes also encompass the farming system, involving environmental sustainability, animal welfare, and characteristics such as free-range/welfare-friendly/local/autochthonous type [7]. Studies indicate that consumers are convinced of a significant connection between the local breeds and the terroir [10], value this unique combination, and believe in the specific quality characteristics testifying to its excellence from among food production. This implies that a higher grade of image value is linked to better quality perception, and that consumers would be willing to pay more for the product that is perceived as valuable to them [9]. As a result, the growing consumer awareness towards extrinsic features stimulates the development of new or niche markets, offering unconventional meat products targeted at consumers sensitive to the above-mentioned features. Analysis of purchasing choices and “willingness to pay more” shows that consumers value the information that pigs were fed local feed (farmers’ own feed) more than information about the animal breed itself [11], which further justifies our steps to develop a health-promoting feed additive based on local herbs and fruits. This novel conception we are working on [12,13]—combining a native pig breed, local alternative protein and energy sources, and functional feed additives—may be an interesting alternative for pig farmers focusing on both caring for pig health and the production of meat with health-promoting value without compromising performance.

With the improvement of people’s living standards and the access to knowledge, consumers’ perception of meat quality and safety issues is highly connected with health and nutritional values. Improved dietetic balance of fatty acids composition of meat, including a higher level of PUFA, especially n-3 PUFA, is one of the leading directions [14]. Enriching pig diets with n-3 polyunsaturated fatty acids (PUFAs) allows for meat with a higher content of these acids [15], which is beneficial from dietetic point of view [16]. However, PUFA n-3 susceptibility to oxidation is high and leads to the formation of toxic fat oxidation products, and excessive free radicals react with membrane lipids or proteins to induce cellular and tissue damage, which can alter not only the health but also the flavor and nutritional value of pork [17,18]. Therefore, such feeding should be combined with the addition of antioxidants to the feed, which may limit the problem of worsening the color, texture, aroma, or flavor by increasing pork oxidative stability [19]. Protection against oxidation can be achieved by adding synthetic antioxidants or natural products to pig feed, such as plants (herbs and fruits) rich in phenolic compounds with a broad spectrum of antioxidant activity. The latter option is not commonly used in large-scale pig production (except for plant extracts), but it can be used in extensive production systems, such as those for slow-growing or organically raised pigs. In our research, we focused on finely ground dried herbs or fruits (not extracts), as this is a cheaper option and can be obtained on the farmer’s own farm. In order to reduce the cost of feeding, low-tannin faba bean seeds, which do not require expensive thermal processing, were added to the pigs’ diet as the local protein source, as well as the by-product of oil pressing—rapeseed cake. This is consistent with sustainability as a publicly accepted model of animal production that cares about the environment and product quality, but also about the farmer’s finances [1]. The feeding strategy combining the use of a local protein source and local natural antioxidants with native pig breeds is in line with the principles of circular farming, as well as local production “from farm to fork”, and reducing transport emissions. In addition, interest for and use as a feed supplementation of the plants available and familiar in a given region will give an interesting added value to the obtained animal products. With increasing competition on the food market, the strategy we propose creates an opportunity for producers willing to differentiate their products, serving specific products with some added value adapted to local conditions [11].

The novelty of our research is the use of a composition of local natural sources of n-3 PUFAs together with local natural antioxidants, introduced to feed containing alternative protein sources intended for native pig breeds, in order to protect the health status of pigs used for producing high-PUFA n-3 dietary meat. We hypothesized that flaxseed-based diets supplemented with locally sourced dried plants would improve antioxidant and immune response without adversely affecting growth performance, metabolic health or meat quality. The study aimed to evaluate the effects of feeding strategies based on flaxseed combined with locally available dried herbs, or fruits, or herbs with beetroot, on growth performance, carcass traits, meat quality, fatty acids profile, and laboratory health parameters associated with the antioxidant and immune status of the slow-growing Pulawska pig breed, fed diets based on alternative protein sources (local and by-product).

## 2. Materials and Methods

### 2.1. Experimental Design, Animals and Diets

The study involved 40 fattening pigs (20 male and 20 female) of the Pulawska breed, weighing approximately 30 kg and possessing the same RYR1 (NN) genotype. The pigs were kept in a control station, which allowed the animals to be kept in individual pens bedded with straw, meeting the animal housing conditions in terms of pen area and ambient temperature, and lighting was provided by natural daylight. All animals were fed the same grower feed mixture until they reached 90 kg of body weight, then for the final rearing period (90–135 kg BW), they were randomly assigned to four feeding groups of 10 pigs each. When dividing the pigs into groups before differentiation of feeding treatment, sex and initial body weight were taken into account; animals from the same litter were not placed in one group. The finisher period lasted on average 84.4 days in each group. The feed mixtures in each group were isoenergetic and isonitrogenous, covering the pigs’ nutritional requirements [20] (Table 1). In the control group, the fattening pigs received a feed mixture without additives. The next three experimental groups received a feed mixture supplemented with 3% extruded flaxseed served together with 2% dried herbs (group H), or 2% dried fruit (group F), or 2% herbs combined with dried beetroot (group HB). The experimental design and the type of feed additives for this experiment were chosen based on the results of our previous preliminary studies. The results obtained in those studies indicated that combining flaxseed together with some kind of antioxidant provides higher n3 PUFA content in meat and better protection against the oxidation of n3 PUFAs at the same time, which allows for greater benefits and may have practical implications.

Dried plants were finely ground before being introduced into the feed mixtures. The herbal additive (H) included dried plants rosemary, thyme, sage, hop and caraway seeds; the fruit additive (F) included dried rose canine, sea buckthorn, elderberry, rowanberry and caraway seeds; the herbal-beetroot additive (HB) included dried rosemary, thyme, sage, hops, caraway seeds and red beetroot. The dried plant materials, before being selected for this research, were analyzed for their nutrient and active ingredient content, and their antioxidant properties were also tested using the DPPH test [13]. After the animals reached a final body weight, they were subjected to the same standard procedure of slaughter, including fasting and free access to water before slaughter. The pigs were transported about 100 m on a transport cart to the slaughterhouse, which is part of the experimental station complex. Slaughter was performed using electric tongs (Koma STZ-6i, PPHU KOMA Sp. z o.o, Świdnica, Poland) with parameters consistent with applicable standards.

### 2.2. Chemical Analysis of Feed, Blood and Meat

The blood samples were centrifuged at 3500× *g* for 15 min to obtain serum, which was frozen (−20 °C) until analysis. The biochemical parameters were colorimetrically measured using test Cormay’s kits (Lublin, Poland) and a BS-180 biochemical analyzer (Shenzhen Mindray Biomedical Electronics Co. Ltd., Shenzhen, China). The levels of total protein, albumin, urea, total cholesterol, high-density lipoprotein (HDL) and low-density lipoprotein (LDL) fractions, and triglycerides (TGs) were determined. The activity of alkaline phosphatase, aspartate aminotransferase, and alanine aminotransferase enzymes was also analyzed.

Concentrations of blood immunoglobulins IgA (cat. no. EP0076), IgG (cat. no. EP0084), IgM (cat. no. EP0085), IL-6 (cat. no. EP0099), IL-8 (EP0176), haptoglobin (EP0070) and Lysozyme (EP0107) were determined using commercially available ELISA kits (Wuhan Fine Biotech Co., Ltd., Wuhan, China) and measured with an ELISA Sun-rise™ microplate reader (Tecan Austria GmbH, Grödig, Austria).

Antioxidant status in the fattening pigs was assessed in blood by measuring ferric reducing antioxidant power (FRAP), superoxide dismutase (SOD) and catalase (CAT) activities, reduced glutathione (GSH) concentration, and oxidative stress markers, including malondialdehyde (MDA) and lipid hydroperoxides (LOOH). LOOH was determined using the FOX2 method according to Södergren et al. (1998) [22]. MDA was measured according to Esterbauer and Cheeseman (1990) [23], FRAP and GSH following Benzie and Strain (1996) [24] and Jollow et al. (1974) [25], respectively. SOD and CAT activities were determined according to Beauchamp and Fridovich (1971) [26] and Aebi (1984) [27]. The same assays were applied to meat homogenates for MDA, LOOH, GSH, SOD, and CAT determination.

Fatty acid profile of the finisher diet was determined by gas chromatography using a SHIMADZU GC-2010 Plus apparatus (Shimadzu Corporation, Kyoto, Japan) Column Rtx2330, 105 m, 0.32 mm, 0.2 μm) according to P015 issue 2 dated 01.03.2016. Samples were mixed, and lipids were extracted using a chloroform–methanol solution (2:1) [28]. After evaporation under a stream of nitrogen and saponification (80 °C, 0.5 N NaOH in methanol), the samples were subjected to methylation using a boron trifluoride/methanol reagent [29]. Fatty acid methyl esters were extracted with hexane and separated using a polar capillary column (105 m × 0.32 mm inner diameter, 0.2 μm film thickness, Rtx-2330; Restek, Bellefonte, PA, USA) on a GC-2010 Plus gas chromatograph (Shimadzu). The temperature program was as follows: column temperature from 60 to 120 °C at 20 °C/min, then from 120 to 240 °C at 3 °C/min; injector temperatures were set at 250 °C. The detector FID was operating in full scan mode. The temperature of the ion source was 250 °C. Helium was used as the carrier gas. The analysis was performed using a standard fatty acid solution (Merck, Darmstadt, Germany) processed in the same way as the samples. The results were expressed as grams per 100 g of total fatty acids detected.

The fatty acid profile of the meat was estimated using the same method as described above for the feed, except that the samples stored at −20 °C were mixed, and the quadrupole electron ionization (70 eV) mass spectrometer was operating in full scan mode. The determined fatty acids were expressed in g per 100 g of all determined acids.

The content of dry matter, crude protein and crude fat in meat was determined according to the Official Method of Analysis of AOAC (2009) [30]. Meat lipid oxidation (TBARS) was determined by method P025 Issue 2, 18/12/2019 based on Pikul et al. (1989) [31].

### 2.3. Carcass and Meat Quality Analysis

After the carcasses had been cooled at 4 °C for 24 h, the dissection analysis of the right side of carcasses was conducted. Carcass quality was evaluated according to standard methods used at the Pig Performance Testing Stations [32].

The samples of *longissimus lumborum* (LLM) muscle were taken from the area between the last thoracic vertebra and the first lumbar vertebra for further analysis—which are described in detail in Szyndler et al., 2025 [13]. In general, the following qualitative characteristics were assessed in the meat (*m. longissimuss lumborum*): (1) color parameters (*L*, *a*, *b*) including lightness (L*), redness (a*), and yellowness (b*) measured in fresh samples (24 h after slaughter) with a Minolta CR-310 colorimeter (Konica Minolta, Tokyo, Japan); (2) Warner–Bratzler shear force (and toughness—shear energy) performed on cooked meat; (3) Texture analysis (hardness, cohesiveness, chewiness, resilience and springiness) performed using texture analyzer TA-XTplus (Stable Micro Systems, Godalming, UK), the resulting data were collected and calculated by Texture Expert software version 1.20. Chewiness (the energy required to grind/chew a solid product) was calculated using hardness, cohesiveness and springiness data.

### 2.4. Statistical Analysis

Data were analyzed by one-way analysis of variance (ANOVA) using the Statistica version 13.3 software package (StatSoft Inc., Tulsa, OK, USA) [33]. Before analysis, the data were checked for normality using the Shapiro–Wilk test and for homogeneity of variances using Levene’s test. When the overall ANOVA effect was significant, differences between group means were evaluated using Tukey’s post hoc test to correct for multiple comparisons. Differences between mean values were considered statistically significant at *p* ≤ 0.05.

Each pig served as an experimental unit (n = 10 per group). The sample size used in the present study was determined based on an a priori power analysis using Cohen’s effect size (f) estimated from a previous experiment conducted under comparable conditions. The previous study included seven dietary treatment groups (n = 8 per group), and representative biochemical variables exhibiting moderate to large treatment effects (e.g., glucose, ALP, albumin, total protein, and LDH) were used to estimate the required sample size. The analysis indicated that 10 pigs per group would provide statistical power greater than 0.90 to detect biologically relevant treatment effects using one-way ANOVA.

The statistical analyses were performed using the following one-way ANOVA model:

Yij = μ + Di + εij

where Yij is the observed value of the dependent variable, μ is the overall mean, Di is the fixed effect of dietary treatment, and εij is the residual error.

Principal component analysis (PCA) was performed using GraphPad Prism version 10.0.2 for Windows (GraphPad Software) to visualize the distribution of individual animals based on blood immune and antioxidant parameters. The analysis included IgA, IgG, IgM, IL-6, IL-8, SOD, CAT, GSH, FRAP, LOOH, and MDA. Because these variables were expressed in different units and numerical ranges, they were standardized before analysis using the “Standardize” option in GraphPad Prism. The first two principal components were plotted in a PCA score plot, with individual animals shown as points and dietary groups indicated by different colors. The percentage of variance explained by each component was displayed on the corresponding axis. PC1 and PC2 explained 47.2% and 22.7% of the total variance, respectively. PCA loading coefficients for PC1 and PC2 were extracted to identify the variables contributing most strongly to the separation of animals along the first two principal components.

## 3. Results

Analyzing the fattening characteristics of the Pulawska pigs (Table 2), it was found that the animals fed the diet supplemented during the finisher period with flaxseed together with dried herbs or fruit showed similar daily weight gain and feed utilization (*p* > 0.05). Dressing percentage, weight of the main carcass cuts, backfat thickness and meatiness were similar in all groups. Regarding meat quality traits, no differences were found between the groups in meat acidity, meat color, or the content of basic nutrients (*p* > 0.05) (Table 2).

The content of PUFA n-3 in meat was increased by more than two times (*p* < 0.0001) in groups fed the feed mixture with the tested supplements—including flaxseed, which was confirmed by the significantly lower (by 57–64%) PUFA n6/n3 ratio in meat (*p* < 0.0001) (Table 2). The level of PUFA n-6, MUFA and SFA remained unchanged (*p* > 0.05).

**Figure 1:**
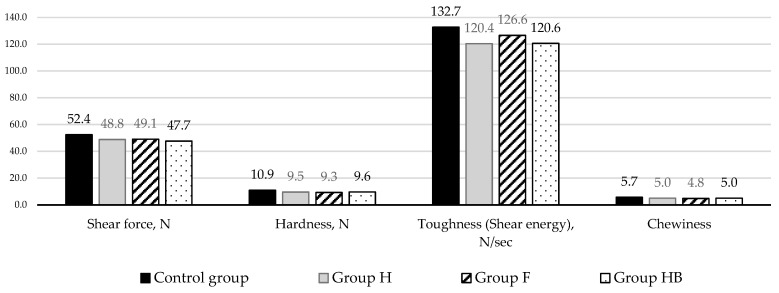
The results of the Warner–Bratzler shear force and texture profile analysis (TPA) in meat (*longissimus* m.). Groups: Control—basal diet without additives; H—3% extruded flaxseed + 2% dried herbs; F—3% extruded flaxseed + 2% dried fruit; HB—3% extruded flaxseed + 2% dried herbs with beetroot.

The meat quality results presented in Figure 1 are interesting, because despite the lack of significant differences (*p* > 0.05), it can be noticed that the meat of the pigs receiving flaxseeds and dried herbs or fruit in their feed was characterized by more favorable values of shear force (by 6.3–8.9%), hardness (by 11.9–14.7%), toughness (by 4.6–9.3%) and chewiness (12.3–15.8%).

To assess the effect of the studied nutritional factors on the blood parameters of Pulawska pigs, analyses were performed on the lipid (CHOL, TG, HDL, LDL), hepatopancreatic (ALT, AST, ALP, GLU, ALB), renal (CREAT, UREA, TP, Ca, P, Mg), and bone (Ca, P, ALP, ALB) blood profiles (Table 3). Adding flaxseed to the feed mix, along with dried herbs (group H), fruit (F), or herbs and beetroot (HB), had no significant effect on the tested biochemical parameters of the blood of Pulawska pigs.

The studied nutritional factors, i.e., dried plants (herbs, fruit, herbs with red beetroot), significantly influenced the redox status of blood and tissues of Pulawska pigs receiving a feed mixture with 3% ground flaxseed, compared to the control group (Table 4). In the blood of pigs receiving the feed mixture with flaxseed and dried herbs (H), higher activity of catalase and glutathione peroxidase (almost twice as high), and superoxide dismutase (over 7%) as well as the level of FRAP (total antioxidant potential) (over 30%), was found compared to the control group (*p* < 0.0001). It should be noted that the activity of these enzymes (SOD, CAT) and GSH content was also significantly higher in the blood of the pigs from groups F and HB compared to the animals from the control group; however these differences were slightly smaller. In the blood of the pigs receiving a feed mixture containing flaxseed and dried herbs (H), we found significantly lower levels of LOOH and the end-product of lipid peroxidation—MDA—compared to the control group (by 16% and 28%, respectively) (*p* < 0.0001). Significantly lower (*p* < 0.0001) levels of MDA were also observed in the pigs from groups F and HB compared to the control group (by 22% and 11%, respectively); however, the LOOH in group HB remained significantly high.

Flaxseed with dried herbs (H), dried fruit (F), or herbs and beetroot (HB) added to feed mixtures for fattening pigs inhibited the formation of lipid peroxidation products, MDA and LOOH, in meat compared to the control group (*p* < 0.0001), and contributed to an increase in the activity of CAT, SOD (groups H and HB) and GSH content (group H only) (Table 4). It should be noted, however, that the highest differences, reaching 43% for CAT, 9.5% for SOD, 61% for GSH, 34% for MDA, and 31% for LOOH, were observed in the meat of pigs receiving flaxseed and dried herbs (group H) compared to the control group. Due to the presence of active substances in the dried herbs and fruit, the meat of pigs receiving a 3% flaxseed supplement in their feed (groups H, F and HB) did not demonstrate poorer resistance to oxidative processes (TBARS in the experimental groups was similar to the control group, *p* = 0.922) occurring during 2 months of frozen storage.

The stimulation of antioxidant processes caused by the inclusion of dried herbs (H) or dried fruit (F) in the feed mixes for the fattening pigs also resulted in improved parameters of the proper functioning of the immune system (Table 5). Compared to the control group, all additives significantly increased the IgA in the pigs’ blood (*p* < 0.0001) compared to the control group; however, the IgG levels were significantly higher only in the blood of the pigs receiving dried herbs (H) or fruit (F) compared to the control pigs, not in the pigs receiving dried herbs and beetroot (HB). A similar relationship was observed for lysozyme, which is part of the innate immune system. The IgM was higher than in the control group, only in the case of the pigs fed a diet with herbs (H) (*p* < 0.0001). The levels of pro-inflammatory interleukins (IL6, IL8) and acute phase proteins (haptoglobin) were also determined in the pigs’ blood (Table 5). IL8 secretion is increased by oxidative stress, which causes the formation of inflammatory cells and induces a further increase in oxidative stress mediators. Furthermore, the literature data indicate that IL8 levels are associated with obesity. The addition of flaxseed and dried herbs (group H) to the feed mixture reduced the levels of both interleukins (IL6 by 12.5, and IL8 by 13.9%) in the pigs’ blood (*p* ≤ 0.05), compared to the control group. Flaxseed combined with dried fruit (group F) had no effect on interleukin levels. Compared to the control group, significantly lower HP levels were noted in the animals from groups H and HB (*p* = 0.0094).

**Figure 2:**
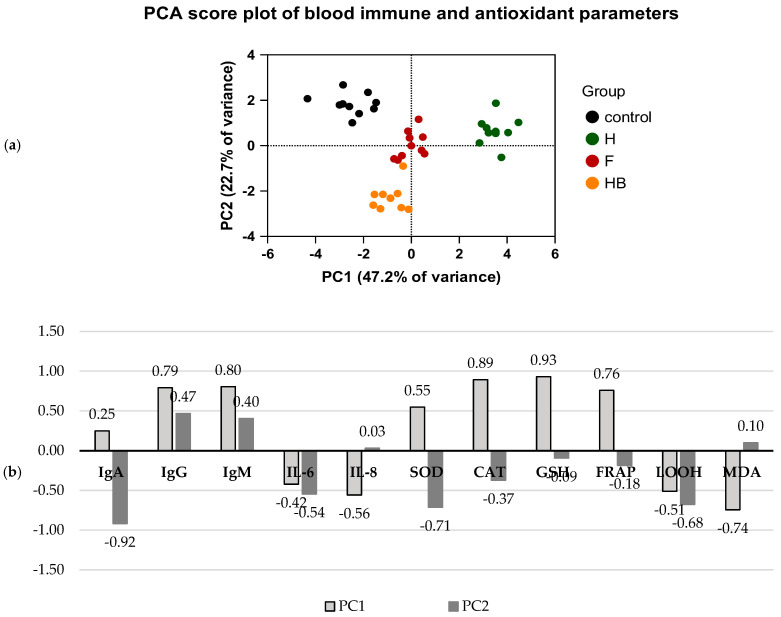
(**a**) A principal component analysis score plot based on the immune and antioxidant blood parameters of the Pulawska pigs; (**b**) the loading coefficients of blood immune and antioxidant parameters on the first two principal components. PC1 and PC2 explained 47.2% and 22.7% of the total variance, respectively. Groups: Control—basal diet without additives; H—3% extruded flaxseed + 2% dried herbs; F—3% extruded flaxseed + 2% dried fruit; HB—3% extruded flaxseed + 2% dried herbs with beetroot.

Principal component analysis performed on blood immune and antioxidant parameters revealed a clear separation of dietary treatments (Figure 2a). The first two principal components explained 69.9% of the total variance, with PC1 accounting for 47.2% and PC2 for 22.7%. The interpretation of the PCA score plot was supported by the loading coefficients for PC1 and PC2, which are presented in Figure 2b. PC1 was mainly positively loaded by antioxidant defense markers, including GSH, CAT, FRAP and SOD, as well as immune parameters IgG and IgM. In contrast, negative PC1 loadings were observed for lipid peroxidation markers, particularly MDA and LOOH, and for IL-8. Thus, positive PC1 scores reflected a more favorable immune-redox profile, characterized by higher antioxidant potential and immunoglobulin levels together with lower lipid peroxidation. The control group was clearly separated from the supplemented groups and was located mainly on the negative side of PC1, which was consistent with higher lipid peroxidation markers and lower antioxidant potential. In contrast, group H was positioned on the positive side of PC1, reflecting higher CAT, GSH, FRAP, SOD, IgG, and IgM values. Group F occupied an intermediate position between the control and the other supplemented treatments. Group HB was separated mainly along PC2, which was primarily associated with IgA, LOOH, SOD, and IL-6, showing negative loadings, and with IgG and IgM, showing positive loadings. This indicates that PC2 contributed mainly to the separation of animals with a distinct IgA- and IL-6-related response. Overall, the PCA results (Figure 2) supported the univariate analyses and indicated that the combination of flaxseed with dried herbs produced the most distinct improvement in antioxidant and immune status. In contrast, the herb–beetroot treatment showed a different immunological profile, mainly related to IgA response.

## 4. Discussion

One of the most common civilizational diseases is cardiovascular disease, both in developed and developing countries [34]. According to WHO guidelines (2022) [35], reducing the risk of cardiovascular disease can be achieved by increasing the consumption of polyunsaturated fatty acids (PUFAs), especially n-3 PUFAs [36]. At the same time, actions should be taken to limit the formation and undesirable effects of free radicals, which arise as a result of PUFAs’ susceptibility to oxidation. Oxidative stress can contribute to endothelial dysfunction; cell death; oxidative damage to amino acids and proteins, including mitochondrial DNA; lipid peroxidation; and the inhibition of enzyme activity and transport proteins, and induce the expression of cyclooxygenase, which activates the inflammatory potential [37]. Given that pork is currently the second most frequently consumed meat in the world [38], and therefore a popular and quantitatively significant source of fatty acids in the human diet, as well as the known significant relationship between dietary fatty acid profiles and the tissue fatty acid composition in pigs [39,40], we believe it is justified to investigate pig feeding strategies that will improve the dietary value of pork. The feed additives we developed and tested, composed of a source of n-3 PUFA (flaxseed) and a source of antioxidants (locally available dried herbs, fruits, or herbs with beetroot), most closely meet these expectations. In Central European conditions, including Poland, popular plants whose fruits are rich in phenolic compounds include rosa canina, sea buckthorn, elderberry, and rowanberry, while local herbs include rosemary, thyme, sage, and hops. Dried herbs from these plants were tested in our earlier research to confirm their antioxidant potential [13]. The herbs used in the present study contained polyphenols ranging from 3.5% (sage), through 4.8 (thyme), to 6.5% (rosemary), as the content of total hydroxycinnamic acid derivatives expressed as a percent of rosmarinic acid. Dried fruit contained polyphenols expressed as a percent of pyrogallol, ranging from 1.6% (red beetroot), through 3–5% (hop, sea buckthorn, elderberry, and rosa canina) to 16.4% (rowanberry).

When assessing the impact of the studied feeding strategies on the production parameters of fattening pigs of the native Pulawska breed, no significant effect was found on body weight gain, feed conversion ratio (FCR), carcass quality (meat content, fat content, and weight of main carcass cuts), or meat quality. This is a very satisfactory result, confirming that the proposed diets do not negatively impact fattening and farmer profits, which is the basis for interest in the research results in production practice. Similar results, in the scope of carcass and meat quality, were observed in our previous studies conducted on pigs of another native breed (Zlotnicka Spotted), fed the same feed supplements [13], where not only the quality of raw meat from the *longissimus* m. but also the *semimembranosus* m. and meat from the *longissimus* m. prepared as a raw matured product were analyzed.

As a descriptive observation, it can be mentioned that despite the lack of significant differences (*p* > 0.05), the meat of pigs receiving flaxseed with dried herbs or flaxseed with dried fruits in their feed was characterized by more favorable organoleptic values of shear force (by 6.3–8.9%), hardness (by 11.9–14.7%), toughness (by 4.6–9.3%) and chewiness (12.3–15.8%). This is an interesting observation; however it needs further research. As expected in our experiment, pigs receiving flaxseed in their feed showed beneficial changes in the fatty acid profile of meat, which translated into a significantly higher n-3 PUFA content and a much-narrowed n-6/n-3 PUFA ratio. The favorable impact of the proposed feed supplements for native breed pigs was observed also in the atherogenic and thrombogenic indices, and the ratio of hypocholesterolemic to hypercholesterolemic acids. This is consistent with changes observed in another native breed, Zlotnicka Spotted, fed the same diet [13]. In studies of other authors, the use of extruded flaxseed in feed mixtures also affects the deposition of PUFA in meat and subcutaneous fat [41,42]. The effect of lowering SFA content, increasing PUFA n-3, and lowering the PUFA n-6/n-3 ratio, as well as desired changes in thrombogenic index in loin meat was observed by Klimiuk et al. (2023) [43], feeding pigs with a mixture containing flaxseed oil and the addition of 3% of thyme. Another plant feed additive—dried bergamot pulp—caused a lowering effect on the PUFA n-6/n-3 ratio in loin pig meat and salami, and on trombogenic and atherogenic indexes measured in salami [44].

The results obtained in our experiment after analysis of the pigs’ blood and muscle suggest that the inclusion of dried herbs or dried fruits in the feed mixture inhibits oxidative processes occurring in the bodies of Pulawska pigs fed with flaxseed. However, this inhibition was most intensely reflected when dried herbs were used. The beneficial effects of the tested diets were reflected mainly in antioxidant parameters. The inclusion of dried herbs, fruits, or herbs with beetroot enhanced the antioxidant defense system in blood and muscle, as shown by increased activities or concentrations of CAT, SOD, GSH, and FRAP, together with reduced levels of lipid peroxidation products such as MDA and LOOH. This response suggests that the bioactive compounds present in the mixtures of chosen dried local plants were able to counteract oxidative processes associated with a PUFA-enriched diet. Among the tested combinations, flaxseed with dried herbs produced the most pronounced improvement in the redox profile, which was evident both in blood and in *longissimus* muscle. Klimiuk et al. (2023) [43] showed that thyme supplementation in diets with extruded flaxseed affected the antioxidant and lipid profile of blood and tissues, including changes in total cholesterol concentration. Moreover, thyme supplementation increased SOD and CAT activity, and reduced FRAP and LOOH values. Importantly, as in the present experiment, no adverse changes in hepatic markers, including ALT and AST, or renal indicators, such as creatinine and urea, were observed. Similarly, studies by Klimiuk et al. 2023 [45] and Czech et al. 2023 [46] indicate that the inclusion of thyme in PUFA-enriched diets may support antioxidant status in blood and muscle and modulate lipid metabolism in fattening pigs. In addition to thyme, other components of the herbal mixture may also have influenced the antioxidant response. Rosemary is particularly rich in phenolic diterpenes and phenolic acids, which are associated with antioxidant activity [47]. In broilers [48], dietary supplementation with an ethanol extract of rosemary increased the activity of SOD and CAT, as well as GSH concentrations, whilst reducing MDA and LDL cholesterol levels. Similarly, in hyperlipidemic rats, rosemary preparations increased SOD and total antioxidant capacity and reduced serum CHOL, TG, and lipid peroxidation [49].

The presence of dried herbs in feed mixture containing flaxseed as a PUFA n-3 source limited the formation of primary peroxidation products, which is why both LOOH and MDA decreased. However, the mixture of dried fruits was less effective—MDA decreased, but LOOH did not decrease to the same extent. Fruits contain high levels of polyphenols, but they might be less stable during the drying process; some compounds have lower bioavailability, and their antioxidant activity depends on intestinal metabolism. Therefore, the fruit mixture was sufficient to increase total antioxidant potential, but insufficient to fully inhibit the formation of lipid hydroperoxides. Group HB demonstrated the most complex response: improved antioxidant system activity (FRAP, CAT, SOD) and reduced MDA did not translate into a reduction in LOOH in blood. This suggests that the addition of red beetroot either changed the peroxidation process or an interaction between the dried herbs and beetroot occurred, but the mechanism cannot be clearly identified based on the obtained data.

Studies have shown that the use of flaxseed and feed additives of phytogenic origin (rosemary, thyme, sage, hop, caraway seeds, rose canine, sea buckthorn, elderberry, rowanberry, beetroot) may influence immune system parameters in animals, although data for pigs remain limited. The present study demonstrated increased serum IgA concentrations in all groups receiving tested supplements, while IgG levels were elevated in flaxseed groups supplemented with dried herbs (H) or fruits (F), except the group receiving dried herbs with beetroot (HB). Chen et al. (2017) [50] reported that supplementation of sow diets with flaxseed oil increased IgG and IgA concentrations in the blood, colostrum, and milk of sows, as well as in the plasma of piglets. These observations support the hypothesis that flax-derived products may positively modulate humoral immunity. Interestingly, a significant increase in IgM concentration was observed in the pigs receiving the diet supplemented with flaxseed and dried herbs (H) only. Comparable results were reported by Xu et al. (2022) [51], who found that a polyphenol-rich herbal mixture increased serum IgM levels in weaned piglets, while simultaneously improving antioxidant status. Therefore, the enhanced IgM response observed in the present study may be associated with the high content of polyphenolic compounds present in the herbal blend. These bioactive compounds are known to modulate immune cell activity and may stimulate the early stages of humoral immune responses, resulting in increased IgM production. The immunomodulatory potential of herbal additives is also supported by the study of Liu et al. (2022) [52], who demonstrated that dietary rosemary supplementation increased the levels of T-AOC, GSH-Px, SOD, CAT, IL-2, IgA, IgG, and IgM while reducing serum MDA concentrations in broiler chickens. Taken together, these findings suggest that the improvement in humoral immunity observed in the herb-supplemented group may be related to the combined antioxidant and immunoregulatory properties of the phytochemicals present in the herbal mixture. In addition to changes in immunoglobulin concentrations, this study also showed that dietary supplementation with flaxseed, in combination with dried herbs or fruits, increased serum lysozyme activity. This observation is significant because lysozyme is a component of the innate immune system and contributes to the formation of the first line of defense against bacterial pathogens [53]. Consequently, the simultaneous increase in the concentrations of certain immunoglobulins and lysozymes suggests that the plant-based supplements under investigation may support both humoral and innate immune mechanisms. However, it must be mentioned that in our studies, we determined the levels of selected laboratory biomarkers that can demonstrate changes, including improvements, in animal health, but the clinical health outcomes or disease resistance were not studied here. The oxidative stress and inflammation are closely linked processes, and lower concentrations of pro-inflammatory markers may indicate improved systemic homeostasis [54,55]. The reduction in IL-8 and haptoglobin (HP) concentrations in the pigs receiving the herbal supplement suggests that the enhanced immune response was not accompanied by excessive inflammatory activation. This finding is particularly relevant because the increased immunoglobulin and lysozyme concentrations observed in the pigs fed the combination of extruded flaxseed and dried herbs were associated with a more regulated inflammatory response, indicating improved immune homeostasis.

Since antioxidant and immune parameters are closely interconnected and were affected simultaneously by the dietary treatments, PCA was used to evaluate whether these changes formed distinct immune-redox profiles, providing an integrated multivariate assessment of antioxidant and immune responses. The PCA allowed us to visualize the overall immunological and redox profiles of individual animals and demonstrated that dietary supplementation induced distinct multifactorial response patterns. The PCA confirmed that the dietary treatments induced distinct immune-redox profiles in the Pulawska pigs. The separation of the control group from all three groups receiving the proposed feed supplements indicates that the inclusion of flaxseed together with dried herbs, or with dried fruits, or with herbs + beetroot affected the overall pattern of antioxidant and immune parameters. Among the tested treatments, the group fed with flaxseed together with dried herbs (group H) showed the clearest separation and was associated with higher antioxidant defense markers and immunoglobulin levels, together with lower lipid peroxidation parameters. This multivariate pattern supports the results of the individual blood analyses and suggests that the combination of flaxseed with dried herbs was the most effective, which may have potential in improving the systemic antioxidant and immune status of pigs.

The presence of bioactive compounds in the herbal and fruit mixtures may have supported metabolic homeostasis [46,56,57], although in the present study this effect was more clearly reflected in antioxidant and immune indices than in standard biochemical parameters. In the present experiment, despite the clear changes in the fatty acid profile of the *longissimus* m., the combined use of flaxseed with dried herbs or dried fruit additives did not adversely affect blood lipid parameters, which may indicate that the level of flaxseed inclusion used in the study was well tolerated by the slow-growing Pulawska pigs, and that the accompanying plant-derived additives did not cause metabolic overload. The absence of significant differences in basic blood biochemical indices suggests that the inclusion of flaxseed combined with dried herbs, or dried fruits, or herbs with beetroot did not adversely affect the metabolic status of the Pulawska pigs during the final fattening period. In the present study, lipid profile parameters, including total cholesterol, triacylglycerides, HDL, and LDL cholesterol, remained comparable among all dietary treatments. Similarly, the activities of hepatic enzymes, including ALT, AST, and ALP, as well as glucose and albumin concentrations, were not significantly modified by the experimental diets. These results suggest that the tested feeding strategies did not disturb lipid metabolism or hepatic function, despite the markedly increased proportion of n-3 PUFA in the supplemented diets. Renal-related parameters also remained largely unchanged in response to the experimental diets. Only urea showed a tendency (*p* = 0.088) towards differences between groups, with numerically higher values in the pigs receiving flaxseed with dried herbs, or with herbs + beetroot, compared with the control group. This tendency may reflect slight differences in protein metabolism or amino acid utilization. However, the lack of significant changes in CREA, TP, and ALB concentrations suggests that the experimental diets did not impair renal function or overall protein status. Consequently, the lack of any significant effect on the key biochemical blood parameters in this study confirms the safety of combining flaxseed with locally available herbal or fruit supplements, although the main beneficial effects appear to be linked more to the activity of antioxidant and immune indices.

The stronger antioxidant response observed in the pigs receiving the herbal mixture is likely due to the specific composition of these herbs, rather than to the total amount of phytochemicals. Rosemary, thyme, and sage are considered rich sources of highly active antioxidant compounds, including rosmarinic acid, carnosic acid, carnosol, thymol, carvacrol, and various flavonoids. These compounds have been shown to effectively scavenge reactive oxygen species, inhibit lipid peroxidation, and stimulate endogenous antioxidant defense systems by activating antioxidant enzymes [58,59,60]. Consequently, that might be a reason a stronger antioxidant response was observed in the presented experiment in the pigs receiving the herbal mixture. However, although the fruit mixture also provided bioactive compounds with antioxidant properties, the qualitative composition of these phytochemicals differed from that of the herbs used in the diets. Similarly, the herb–beetroot mixture may have contained lower amounts of the most biologically active herbal components, which may have reduced the overall antioxidant potential of this supplement. The better response observed in the group receiving dried herbs likely reflects differences in the qualitative profile, bioavailability, and synergistic interactions of the phytochemicals, rather than merely differences in their total concentration.

At the end of manuscript we would like to highlight the limitations of our study. These include the sample size (number of pigs per group = 10 animals), which might seem relatively small, from the point of view of utilitarian use and implementing the experimental results into farm practice. However, the number of animals used in this experiment was determined taking into account the requirements of the Ethics Committee, and the applicable 3R principle (Replacement, Reduction, Refinement). According to the Reduction principle, the number of animals should be limited to the minimum necessary to obtain reliable and statistically valid results, while maintaining adequate study power. In terms of the quality of the statistics and the reliability of the obtained results, 10 pigs per group is correct—the sample size used in the present study was determined based on an a priori power analysis using Cohen’s effect size (f) which indicated that 10 pigs per group would provide statistical power greater than 0.90 to detect biologically relevant treatment effects using one-way ANOVA. The next point which we see as an important limitation is the evaluation of only one supplementation level of tested feed additives, and the lack of molecular analyses supporting the proposed mechanisms. This deficiency resulted from a lack of funding large enough, and the need to limit the scope of analysis. A number of interesting results obtained so far prompt us to plan further research expanding on these missing aspects. The absence of a flaxseed-only treatment group seem to be a limitation; however the literature data and the results of our previous preliminary study (unpublished) indicated that combining flaxseed together with some kind of antioxidant makes the most sense. These kind of complex feed additive provides higher n3 PUFA content in meat and better protection against the oxidation of n3 PUFAs at the same time, which allows for greater benefits and may have higher practical implications, and it was the basis of our choice. The research conducted by our team, aimed at supporting the production of native pig breeds by identifying innovative diets that will not drastically increase feeding costs but provide added value for the products obtained from these animals, needs further detailed study. This will help to further popularize and support this niche type of pig production.

## 5. Conclusions

The presented research evaluated three feeding strategies dedicated for slow-growing native pigs, consisting of the addition of flaxseed to the feed mixture during the final/finisher fattening period, along with dried herbs (dried rosemary, thyme, sage, hop and caraway seeds), or dried fruits (rose canine, sea buckthorn, elderberry, rowanberry and caraway seeds), or a combination of the herbs mentioned above with dried red beetroot.

Based on the conducted analyses, it was found that the tested diets allowed for an increase in PUFA n-3 content in meat, and at the same time inhibited the negative effects of oxidative stress in the pig organisms, and improved their immune parameters, without affecting the fattening efficiency and physico-chemical meat quality traits.

It can be stated that the tested dietary supplementations can be proposed as an element of sustainable pig production, for native (local) pork with dietetic added value, combining it with meat oxidative stability, with the feed additive containing flaxseed together with dried herbs proving particularly effective.

## Figures and Tables

**Table 1:** The composition and nutrient content of the experimental finisher diets (90–135 kg body weight).

Item	Control Group	Group H	Group F	Group HB
Components [%]				
Wheat	13.0	13.0	13.0	13.0
Barley	39.58	36.56	36.56	36.56
Triticale	13.0	13.0	13.0	13.0
Rye	10.0	10.0	10.0	10.0
Rapeseed press cake	8.0	6.0	6.0	6.0
Low-tannin faba bean	14.0	14.0	14.0	14.0
Monocalcium phosphate	0.22	0.22	0.22	0.22
Ground limestone	1.3	1.3	1.3	1.3
Salt	0.22	0.22	0.22	0.22
L-Lysine	0.18	0.2	0.2	0.2
Mineral–vitamin premix	0.5	0.5	0.5	0.5
Dried herbs mixture	-	2.0	-	-
Dried fruits mixture	-	-	2.0	-
Dried herbs and red beetroot mixture	-	-	-	2.0
Flaxseed	-	3.0	3.0	3.0
In 1 kg of feed mixture:				
Dry matter, g	880	881	881	881
Crude protein, g	147	147	146	147
Crude fat, g	26	37.6	37.7	37.6
Crude fiber, g	47.7	51.0	52.8	51.0
Crude ash, g	48.7	49.5	48.6	49.5
Metabolizable energy, MJ *	12.9	13.0	13.0	13.0
Lysine, g	8.30	8.41	8.37	8.41
Methionine + Cysteine, g	5.13	5.06	5.04	5.06
Threonine, g	4.73	4.70	4.66	4.70
Tryptophan, g	1.98	1.99	1.98	1.99
Calcium, g	6.25	6.20	6.17	6.20
Phosphorus digestible, g	2.04	2.01	2.01	2.01
Fatty acids profile (g per 100 g of all estimated fatty acids):				
SFA	24.25	21.51	22.37	14.72
UFA	75.75	78.49	77.63	85.28
MUFA	22.62	25.24	26.03	27.88
PUFA	53.13	53.25	51.60	57.40
PUFA n-6	49.69	36.93	36.54	40.00
PUFA n-3	3.44	16.32	15.06	17.40

* Metabolizable energy was calculated using the equation from Hoffmann and Schiemann (1980) [21]. Groups: Control—basal diet without additives; H—3% extruded flaxseed + 2% dried herbs; F—3% extruded flaxseed + 2% dried fruit; HB—3% extruded flaxseed + 2% dried herbs with beetroot. SFA—saturated fatty acids; UFA—sum of unsaturated fatty acids; MUFA—sum of monounsaturated fatty acids; PUFA—sum of polyunsaturated fatty acids.

**Table 2:** Results of growth performance and chosen parameters of meat (*longissimus* m.) quality.

Item	Control Group	Group H	Group F	Group HB	*p* Value	SEM
Fattening results:						
Final body weight (BW), kg	133.65	134.64	135.20	136.60	0.831	1.109
Average daily weight gain, g	736	744	747	756	0.953	0.012
Feed utilization per 1 kg of BW gain (FCR), kg	3.53	3.55	3.55	3.57	0.998	0.054
Carcass quality measurements:						
Cold dressing yield, %	76.75	77.14	77.03	77.26	0.419	0.110
Average backfat thickness, cm	2.39	2.47	2.47	2.46	0.970	0.069
Loin eye area, cm^2^	61.07	57.53	56.72	57.46	0.345	0.918
Ham, kg	12.76	13.09	13.16	13.12	0.728	0.137
Bacon with ribs, kg	8.10	8.24	8.18	8.34	0.899	0.108
Pork knuckle. kg	1.57	1.61	1.56	1.62	0.536	0.016
Carcass length, cm	85.90	87.00	86.50	87.70	0.584	0.465
Carcass meatiness, %	56.60	56.20	54.60	55.30	0.121	0.325
Meat basic chemical analysis:						
Dry matter content in meat, %	26.11	25.75	25.94	25.72	0.751	0.141
Protein content in meat, %	23.97	23.54	23.53	23.52	0.513	0.124
Fat content in meat, %	2.09	2.43	2.40	2.22	0.612	0.099
Meat color:						
Lightness, L*	50.78	51.18	51.20	49.71	0.728	0.515
Saturation in red, a*	17.94	18.14	17.94	18.52	0.713	0.199
Saturation in yellow, b*	2.67	1.95	2.65	2.15	0.502	0.202
Fatty acids profile:						
SFA	42.32	41.21	41.97	41.54	0.200	0.195
MUFA	46.58	48.59	46.76	47.54	0.159	0.349
PUFA	11.10	10.19	11.26	10.92	0.725	0.347
PUFA n-6	10.69	9.28	10.25	10.02	0.524	0.336
PUFA n-3	0.41 ^a^	0.91 ^b^	1.01 ^b^	0.91 ^b^	<0.0001	0.050
PUFA n-6/n-3	28.29 ^b^	10.20 ^a^	11.10 ^a^	12.02 ^a^	<0.0001	1.437

^a, b^—values within a row with different superscripts differ significantly at *p* ≤ 0.05. Abbreviations: SFA—sum of saturated fatty acids; MUFA—sum of monounsaturated fatty acids; PUFA—sum of polyunsaturated fatty acids. Groups: Control—basal diet without additives; H—3% extruded flaxseed + 2% dried herbs; F—3% extruded flaxseed + 2% dried fruit; HB—3% extruded flaxseed + 2% dried herbs with beetroot.

**Table 3:** The blood biochemical parameters.

Item	Control Group	Group H	Group F	Group HB	*p* Value	SEM
CHOL, mg/dL	97.60	100.90	99.10	100.40	0.928	1.836
TG, mg/dL	42.23	48.00	45.38	48.23	0.830	2.512
HDL, mg/dL	36.72	37.25	35.47	35.96	0.872	0.792
LDL, mg/dL	57.89	59.24	60.82	59.81	0.921	1.469
CHOL/HDL	2.67	2.75	2.80	2.81	0.719	0.044
ALT, U/L	63.00	63.62	58.65	63.67	0.641	1.574
AST, U/L	57.34	53.63	55.85	58.77	0.895	2.375
ALP, U/L	88.20	95.80	84.50	98.60	0.403	3.269
GLU, mg/dL	75.80	72.90	76.70	74.60	0.903	1.828
ALB, g/dL	4.37	4.55	4.34	4.50	0.507	0.055
CREA, mg/dL	1.74	1.71	1.70	1.76	0.901	0.031
UREA, mg/dL	26.76	31.09	28.06	31.98	0.088	0.844
TP, g/dL	7.64	8.11	7.84	8.15	0.339	0.112
Ca, mg/dL	12.85	13.07	12.58	13.49	0.239	0.161
P, mg/dL	10.84	11.01	10.69	10.94	0.985	0.293
Mg, mg/dL	2.66	2.64	2.51	2.82	0.427	0.063
Fe, µg/dL	186.35	188.64	187.43	185.75	0.997	4.668

CHOL—total cholesterol; HDL—high-density lipoprotein; LDL—low-density lipoprotein; TG—triacylglycerides; ALT—alanine aminotransferase; AST—aspartate aminotransferase; ALP—alkaline phosphatase; GLU—glucose; ALB—albumin; CREA—creatinine; UREA—urea; TP—total protein; Ca—calcium; P—phosphate; Mg—magnesium; Fe—iron. Groups: Control—basal diet without additives; H—3% extruded flaxseed + 2% dried herbs; F—3% extruded flaxseed + 2% dried fruit; HB—3% extruded flaxseed + 2% dried herbs with beetroot.

**Table 4:** The antioxidant parameters in blood and meat (*longissimus* m.).

Item	Control Group	Group H	Group F	Group HB	*p*-Value	SEM
BLOOD						
FRAP, µmol/L	14.45 ^a^	19.24 ^b^	15.70 ^a^	16.32 ^a^	<0.0001	0.385
CAT, U/mL	4.69 ^a^	10.87 ^d^	7.56 ^b^	8.47 ^c^	<0.0001	0.367
SOD, U/mL	23.44 ^a^	25.25 ^bc^	25.02 ^b^	25.54 ^c^	<0.0001	0.145
GSH, µmol/L	0.90 ^a^	1.79 ^d^	1.31 ^c^	1.21 ^b^	<0.0001	0.053
MDA, µmol/L	4.31 ^c^	3.10 ^a^	3.34 ^a^	3.82 ^b^	<0.0001	0.101
LOOH, µmol/L	5.88 ^b^	4.95 ^a^	5.50 ^b^	6.94 ^c^	<0.0001	0.148
MEAT						
CAT, U/mL	24.47 ^a^	35.09 ^b^	33.73 ^b^	33.16 ^b^	<0.0001	0.792
SOD, U/mL	127.59 ^b^	139.75 ^d^	123.39 ^a^	130.72 ^c^	<0.0001	1.052
GSH, µmol/L	0.01 ^a^	0.02 ^b^	0.01 ^a^	0.01 ^a^	<0.0001	0.001
MDA, µmol/L	0.17 ^c^	0.11 ^a^	0.13 ^b^	0.13 ^b^	<0.0001	0.004
LOOH, µmol/L	2.63 ^c^	1.82 ^a^	1.95 ^a^	2.12 ^b^	<0.0001	0.056
TBARS, mg/kg	0.44	0.39	0.42	0.40	0.922	0.024

^a, b, c, d^—values within a row with different superscripts differ significantly at *p* ≤ 0.05. FRAP—ferric reducing antioxidant power; SOD—superoxide dismutase; CAT—catalase; GSH—glutathione; MDA—malondialdehyde; LOOH—lipid hydroxyperoxide. Groups: Control—basal diet without additives; H—3% extruded flaxseed + 2% dried herbs; F—3% extruded flaxseed + 2% dried fruit; HB—3% extruded flaxseed + 2% dried herbs with beetroot.

**Table 5:** Blood parameters of immunological status of pigs.

Item	Control Group	Group H	Group F	Group HB	*p*-Value	SEM
IgA, mg/mL	527.10 ^a^	758.79 ^b^	742.02 ^b^	959.07 ^c^	<0.0001	25.478
IgG, µg/mL	29.69 ^b^	50.80 ^d^	38.29 ^c^	22.06 ^a^	<0.0001	1.804
IgM, µg/mL	141.05 ^ab^	223.95 ^c^	148.84 ^b^	123.27 ^a^	<0.0001	7.072
Lysozyme, mg/mL	1.55 ^b^	1.73 ^c^	1.80 ^c^	1.37 ^a^	<0.0001	0.034
IL6, pg/mL	7.26 ^ab^	6.35 ^a^	8.29 ^bc^	8.64 ^c^	0.0004	0.230
IL8, pg/mL	22.05 ^b^	18.99 ^a^	22.19 ^b^	21.41 ^b^	0.0081	0.391
HP, ng/mL	16.60 ^c^	14.18 ^ab^	15.68 ^bc^	13.74 ^a^	0.0094	0.354

^a, b, c, d^—values within a row with different superscripts differ significantly at *p* ≤ 0.05. Ig—immunoglobulin A, G, M; IL—interleukin 6, 8; HP—haptoglobin; Groups: Control—basal diet without additives; H—3% extruded flaxseed + 2% dried herbs; F—3% extruded flaxseed + 2% dried fruit; HB—3% extruded flaxseed + 2% dried herbs with beetroot.

## Data Availability

Data available from the authors upon request to the corresponding author.
